# Mpox (Formerly Known as Monkeypox) in Dermatological Practice: Presentation of a Series of Four Clinical Cases at a Referral Hospital

**DOI:** 10.7759/cureus.116071

**Published:** 2026-09-10

**Authors:** Valeria Contreras Oceguera, Maria de Lourdes Ochoa Campollo, Melissa Guadalupe Hernandez Gonzalez, Mairany Hernandez Sanchez, José Castillo Gutiérrez

**Affiliations:** 1 Internal Medicine, Instituto Mexicano del Seguro Social, Guadalajara, MEX; 2 Dermatology, Hospital Civil Fray Antonio Alcalde, Guadalajara, MEX; 3 Internal Medicine, Hospital General de Occidente, Guadalajara, MEX; 4 General Medicine, Universidad Autonoma de Zacatecas, Zacatecas, MEX; 5 General Medicine, Universidad de Guanajuato, Guanajuato, MEX

**Keywords:** hiv aids, monkeypox, monkeypox diagnosis, monkeypox treatment, monkeypox virus

## Abstract

Mpox (formerly known as monkeypox) has re-emerged globally as a significant public health concern, with dermatology services occupying a key role in early recognition due to its predominant mucocutaneous manifestations. We present a series of four confirmed mpox cases diagnosed in April 2025 at a tertiary dermatology center in Jalisco, Mexico. All cases occurred in men who have sex with men, three of whom were living with HIV. Clinical presentations were heterogeneous, ranging from disseminated vesiculopustular eruptions with systemic symptoms to strictly localized, painful perianal lesions without prodrome. One case was initially misdiagnosed as herpes, underscoring the diagnostic complexity and potential overlap with other sexually transmitted dermatoses. Epidemiologic risk factors included recent unprotected sexual encounters and, in one case, recent travel. Diagnosis was confirmed by PCR and supported by compatible histopathology when performed. This cluster within a short timeframe reflects ongoing community transmission and highlights the importance of maintaining high clinical suspicion, integrating empathetic and stigma-free sexual history-taking, and leveraging epidemiologic clues for early detection. Increasing diagnostic awareness in dermatology practice is essential to mitigate under-recognition, guide timely management, and support containment strategies.

## Introduction

Mpox, formerly known as monkeypox, is an emerging zoonotic infection caused by an orthopoxvirus, which has transitioned from a sporadic, endemic disease in Central and West Africa to a global public health concern since 2022 [1,2]. Although transmission historically involved animal-human contact, recent outbreaks have demonstrated sustained human-to-human transmission, particularly through close physical and sexual contact, with a disproportionately high incidence among men who have sex with men (MSM) [2-4].

The epidemiologic relevance of mpox has persisted beyond the initial 2022 multinational outbreak. According to World Health Organization surveillance, 63,692 laboratory-confirmed cases and 256 deaths were reported across 105 countries between January 1, 2025, and June 30, 2026 [5]. These data demonstrate continued global transmission and reinforce the importance of sustained clinical awareness even outside the initial outbreak period.

Clinically, mpox is characterized by mucocutaneous lesions that classically progress from macules to vesicles and pustules; however, presentations are heterogeneous, and atypical forms confined to genital or perianal regions have been increasingly recognized [2,6]. Systemic manifestations such as fever, lymphadenopathy, and malaise may precede cutaneous findings but can be absent in a subset of patients, particularly those with localized disease [3]. Such variability may lead to diagnostic challenges and misclassification as other sexually associated dermatoses, including herpes simplex virus infection or syphilis, emphasizing the crucial role of dermatologists in timely recognition [2,3,6].

In Mexico, rising mpox cases have been documented intermittently since the 2022 global outbreak, with surveillance demonstrating ongoing transmission [4]. Early identification remains essential to limit spread and initiate adequate supportive care. Given the evolving epidemiologic patterns and potential under-recognition, dermatology services must maintain a high index of suspicion and integrate thorough, stigma-free sexual and travel history-taking into routine practice.

This report describes four confirmed mpox cases diagnosed over a one-month period in a tertiary referral center in Jalisco, Mexico, highlighting clinical heterogeneity and reinforcing the importance of epidemiologic context in dermatologic assessment and early diagnosis.

## Case presentation

Case 1

A 41-year-old man with newly diagnosed HIV presented with a one-month history of a disseminated polymorphous eruption including pustules, umbilicated vesicles, and crusted lesions on the face, trunk, extremities, palms, soles, and oral mucosa. Systemic symptoms included pruritus, fever, and cervical/inguinal lymphadenopathy. He reported multiple male sexual partners and recent unprotected intercourse. The eruption was initially misdiagnosed as herpes simplex infection, delaying appropriate treatment. Skin biopsy was compatible with mpox (Figure 1).

**Figure 1:**
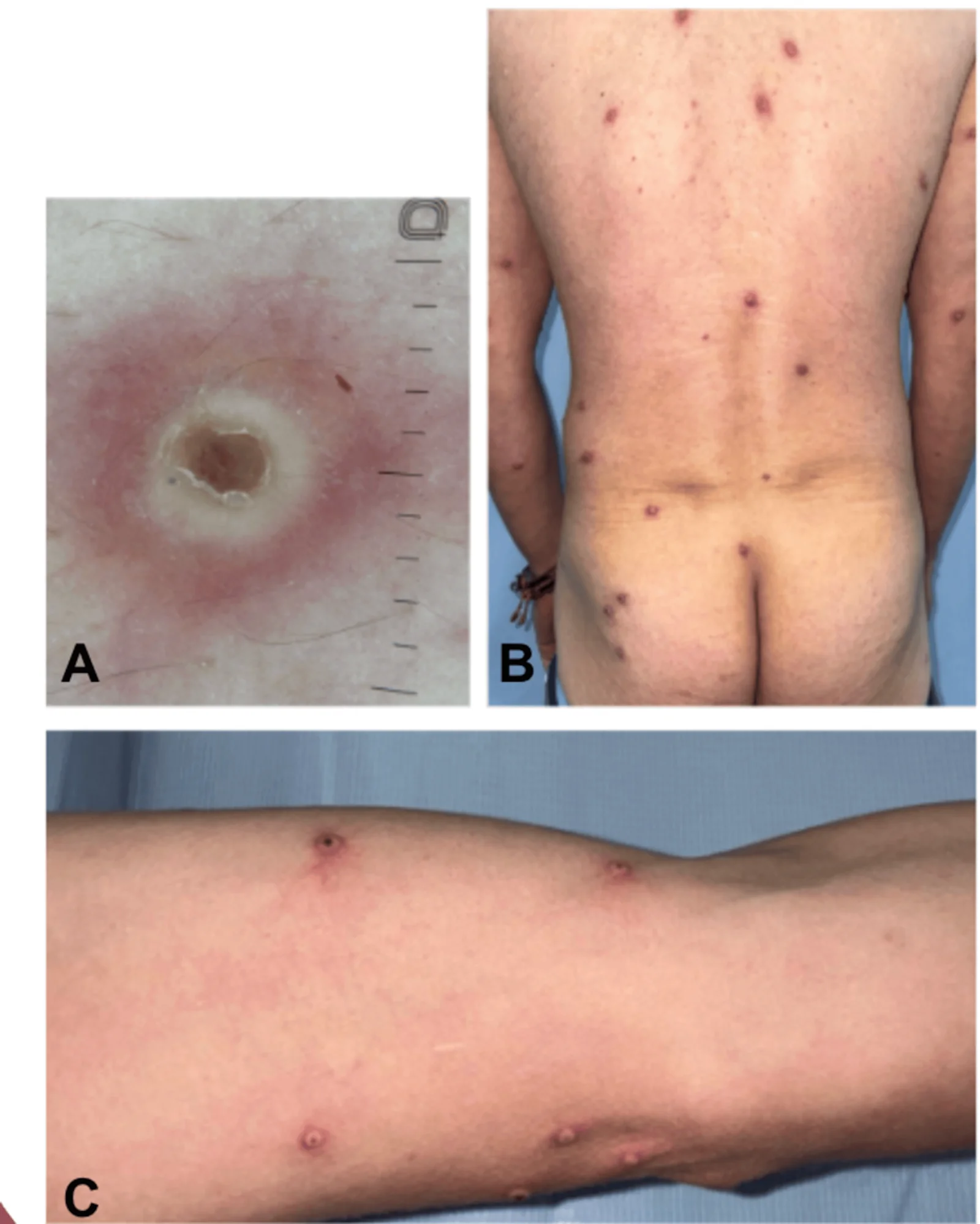
(A) Dermatoscopy showing characteristic form for this lesion, (B) Distribution in the back (C) Lesions in the arm

Treatment

He received symptomatic therapy with antipyretics, oral analgesics, antihistamines, and daily cleansing of lesions. Given his HIV status and extensive mucocutaneous involvement, tecovirimat was initiated.

Response

Within the first week, systemic symptoms improved and lesions began to crust. By day 18 of treatment, nearly all lesions had fully resolved.

Follow-Up

At four weeks, no new lesions were observed and all cutaneous and mucosal findings had healed without scarring or superinfection. His antiretroviral therapy was optimized after diagnosis.

Case 2

A 40-year-old man living with HIV (treatment interruption three months prior) presented with localized painful umbilicated papules forming perianal plaques. Symptoms began one week prior, following prodromal fatigue and diarrhea. He reported more than 100 male sexual partners and recent high-risk anal contact. Biopsy showed findings consistent with mpox (Figure 2).

**Figure 2:**
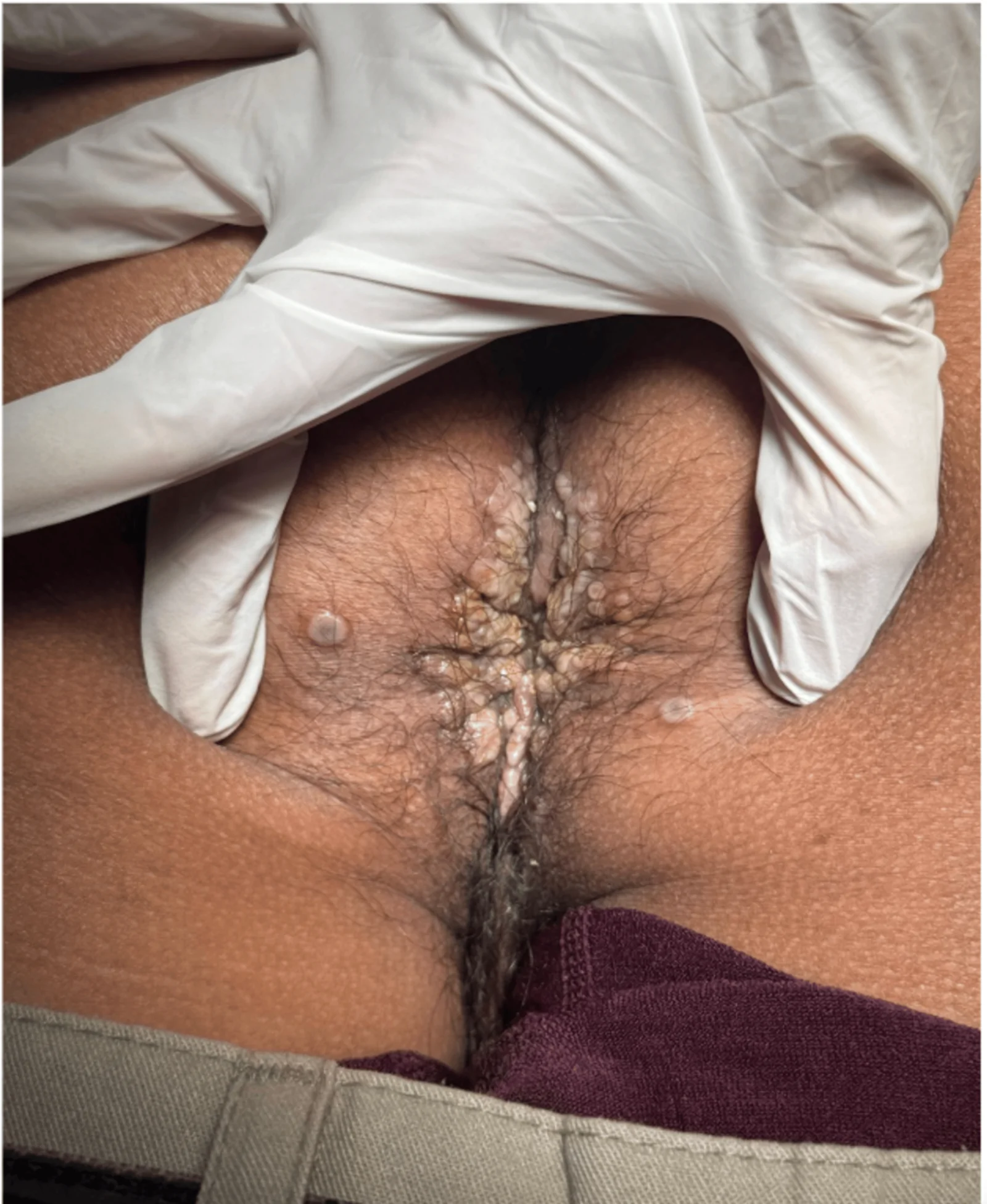
Perianal lesions

Treatment

Because of immunosuppression and severe perianal pain, he received nonsteroidal anti-inflammatory drugs (NSAIDs), topical anesthetics, and hygiene measures. Tecovirimat was started due to clinical severity and uncontrolled HIV.

Response

He experienced significant reduction in pain and inflammation within the first week. Lesions crusted and resolved completely by days 14 to 16.

Follow-Up

At a three-week evaluation, all lesions had healed without functional impairment. He resumed antiretroviral therapy and was referred for sexual health counseling.

Case 3

A 28-year-old man without known comorbidities presented with fever, headache, asthenia, and diaphoresis, followed by oral and pharyngeal lesions causing odynophagia. Days later, non-painful genital and hand lesions developed. He reported sexual contact with men ~15 days before onset and recent travel to Guanajuato. Skin biopsy confirmed mpox (Figure 3).

**Figure 3:**
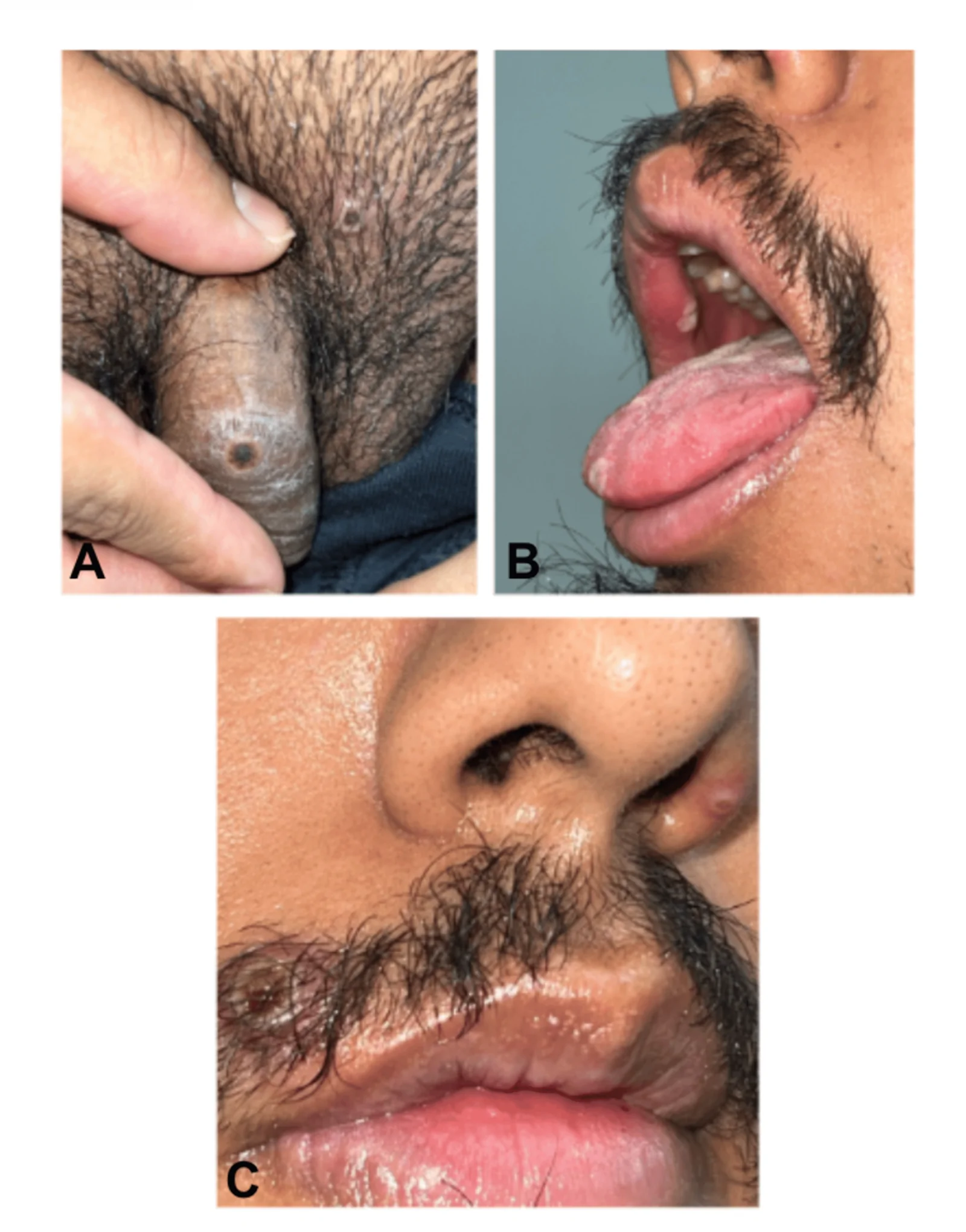
(A) Dorsal penile cutaneous lesion (B) Perioral lesions (C) Lesion on the nasal ala

Treatment

He was managed with oral analgesics, hydration, and topical antiseptics. Due to severe odynophagia, topical lidocaine and short-course corticosteroids were used for symptom control. Antiviral therapy was not indicated as disease remained mild and he was immunocompetent.

Response

Systemic symptoms resolved within five days. Mucosal and cutaneous lesions crusted and detached over 10 to 14 days.

Follow-Up

At a four-week visit, no recurrence or complications were present. Only mild post-inflammatory erythema persisted.

Case 4

A 32-year-old man with HIV on antiretroviral therapy and a recent history of tuberculosis presented with localized painful umbilicated papules forming perianal plaques, without fever or prodromal symptoms. Symptoms began abruptly two days after unprotected sexual contact. PCR and biopsy were consistent with mpox (Figure 4).

**Figure 4:**
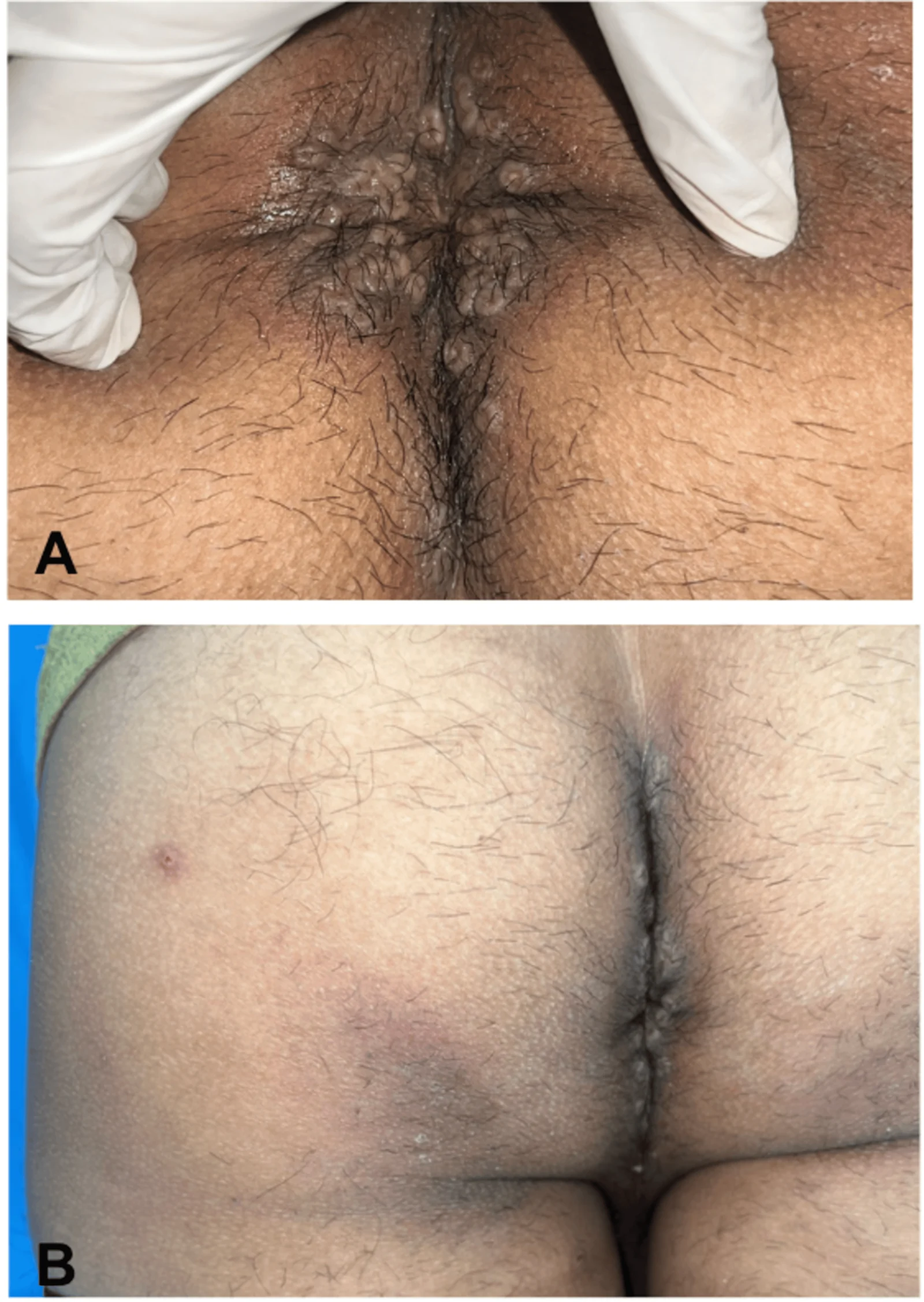
(A and B) Perianal lesions

Treatment

He received analgesics, daily cleansing, and topical lidocaine for pain. Because of his HIV status and history of opportunistic infection, tecovirimat was started early.

Response

Pain improved markedly within 72 hours. Lesions showed rapid crusting and resolution during the following week, without bacterial superinfection.

Follow-Up

By the three-week follow-up visit, lesions had completely resolved with no scarring. His HIV parameters remained stable under ongoing antiretroviral therapy.

## Discussion

This case series illustrates the evolving clinical spectrum of mpox and reinforces dermatology's critical role in early recognition and diagnosis. Since the 2022 global outbreak, mpox has demonstrated sustained transmission outside historically endemic regions, particularly among MSM [1,2]. Similar to international and national reports, the four cases in our series occurred exclusively in MSM, and three patients were living with HIV, a population shown to have higher incidence and occasionally more severe or atypical presentations [2,3,6]. Importantly, one patient in our series was immunocompetent, underscoring that mpox is not restricted to immunocompromised hosts. Compatible mucocutaneous manifestations may occur irrespective of HIV status, and epidemiologic exposure should therefore remain a central component of clinical suspicion [6].

Classically, mpox presents with a systemic prodrome followed by disseminated vesiculopustular lesions [2]. However, consistent with recent literature, our findings highlight significant clinical heterogeneity: two patients presented with isolated, painful perianal lesions, whereas one experienced initial mucosal involvement with odynophagia preceding the development of cutaneous findings. These atypical presentations align with recent evidence describing localized anogenital disease and mucosal-first involvement in sexually acquired mpox, which can mimic other sexually transmitted infections such as herpes simplex virus infection or syphilis [2,3,6]. One of our cases was initially misdiagnosed as herpes, underscoring how mpox can masquerade as other dermatoses, potentially delaying appropriate management and containment measures.

Herpes simplex infection, syphilis, and other sexually transmitted infections were therefore considered clinically relevant differential diagnoses in this series. Importantly, the diagnosis of mpox was not established solely through exclusion of these entities but was confirmed by PCR, with supportive histopathologic findings when performed. A uniform laboratory screening panel for every alternative sexually transmitted infection was not consistently documented in the available clinical records. Consequently, these infections are presented as clinically relevant differential diagnoses rather than as uniformly laboratory-excluded conditions.

Epidemiologic information remains essential for diagnosis. All patients reported recent sexual exposure, consistent with reports identifying close and sexual contact networks as important drivers of transmission [2-4]. Prompt PCR confirmation and histopathologic assessment when performed supported the diagnostic process. The identification of four confirmed cases within a single month at our center suggests ongoing regional transmission and potential under-recognition, consistent with continued surveillance reports in Mexico and internationally [4,5].

Vaccination represents an additional component of mpox prevention. Vaccines based on vaccinia virus, including Modified Vaccinia Ankara-Bavarian Nordic (MVA-BN) and LC16m8, are recommended by the World Health Organization for individuals at increased risk of exposure according to epidemiologic circumstances, individual risk, vaccine availability, and national public health policies [7]. Vaccine availability remains heterogeneous among countries. In the United States, JYNNEOS, an MVA-BN vaccine, is recommended for individuals at increased risk of mpox and may also be used for post-exposure prophylaxis when clinically indicated [8]. Although vaccination was not the focus of the present case series, awareness of available preventive strategies complements timely clinical recognition and public health containment.

Our findings reinforce the importance of maintaining a high index of suspicion for mpox in patients presenting with vesiculopustular, mucosal, genital, or anogenital lesions, particularly when epidemiologic risk factors are present. Importantly, such suspicion should not depend exclusively on HIV status or the presence of systemic symptoms. A non-judgmental, detailed sexual history is indispensable for reducing missed diagnoses and stigma-related barriers to care [2,3]. Ongoing physician education and clear clinical protocols are necessary to ensure timely identification and mitigate further spread [2,4,6].

Several limitations should be acknowledged. This report represents a small retrospective case series from a single tertiary referral center. CD4 counts, HIV viral loads, specific antiretroviral regimens, and complete tecovirimat dosing and duration data were not consistently available in the clinical records. Likewise, laboratory testing for alternative sexually transmitted infections was not uniformly documented. Consequently, the present series was not designed to assess associations between immunologic status, specific antiretroviral or antiviral therapies, and disease severity. Nevertheless, these limitations do not affect the confirmed diagnosis of mpox in the reported cases or the primary descriptive objective of illustrating its heterogeneous dermatologic presentations.

## Conclusions

This case series highlights the diverse clinical spectrum of mpox encountered in dermatologic practice, ranging from classic disseminated vesiculopustular disease to localized anogenital presentations with limited or absent systemic manifestations. The predominance of MSM and individuals living with HIV in this series is consistent with reported epidemiologic patterns; however, the occurrence of disease in an immunocompetent patient emphasizes that clinical suspicion should not depend exclusively on immune status.

Early diagnosis relies on recognizing atypical forms, maintaining a broad differential diagnosis, and integrating thorough, stigma-free sexual and travel history-taking into routine assessment. Strengthening clinician awareness, ensuring timely access to diagnostic testing, and promoting preventive strategies, including vaccination when indicated and available, are important for avoiding misdiagnosis, enabling prompt management, and supporting public health containment efforts. Continued surveillance and reporting remain essential to better characterize evolving transmission patterns and the clinical behavior of mpox in diverse populations.
